# 14-3-3 Proteins Regulate Exonuclease 1–Dependent Processing of Stalled Replication Forks

**DOI:** 10.1371/journal.pgen.1001367

**Published:** 2011-04-14

**Authors:** Kim Engels, Michele Giannattasio, Marco Muzi-Falconi, Massimo Lopes, Stefano Ferrari

**Affiliations:** 1 Institute of Molecular Cancer Research, University of Zurich, Zurich, Switzerland; 2 Department of Biomolecular Sciences and Biotechnology, University of Milan, Milan, Italy; The Hospital for Sick Children and University of Toronto, Canada

## Abstract

Replication fork integrity, which is essential for the maintenance of genome stability, is monitored by checkpoint-mediated phosphorylation events. 14-3-3 proteins are able to bind phosphorylated proteins and were shown to play an undefined role under DNA replication stress. Exonuclease 1 (Exo1) processes stalled replication forks in checkpoint-defective yeast cells. We now identify 14-3-3 proteins as *in vivo* interaction partners of Exo1, both in yeast and mammalian cells. Yeast 14-3-3–deficient cells fail to induce Mec1–dependent Exo1 hyperphosphorylation and accumulate Exo1–dependent ssDNA gaps at stalled forks, as revealed by electron microscopy. This leads to persistent checkpoint activation and exacerbated recovery defects. Moreover, using DNA bi-dimensional electrophoresis, we show that 14-3-3 proteins promote fork progression under limiting nucleotide concentrations. We propose that 14-3-3 proteins assist in controlling the phosphorylation status of Exo1 and additional unknown targets, promoting fork progression, stability, and restart in response to DNA replication stress.

## Author Summary

Stalling and collapse of DNA replication forks is an important source of genome instability and has been implicated in early steps of carcinogenesis. The maintenance of stable intermediates upon stalled replication requires the coordinated action of a number of proteins and proper inhibitory control of dangerous enzymatic activities. In this study, we uncover an evolutionarily conserved mechanism through which 14-3-3 proteins modulate the checkpoint-mediated phosphorylation of, and in turn limit the activity of, an exonuclease (Exo1) previously implicated in pathological processing of stalled replication forks and other sensitive DNA intermediates. This represents an unprecedented link in the field of DNA repair and genome stability, providing a molecular rationale to the yet undefined role of 14-3-3 proteins in the maintenance of genome integrity after replication stress. In analogy to Exo1, our data suggest that additional factors at replication forks may be subjected to similar regulation, pointing to the 14-3-3 proteins as central components of the checkpoint triggered in response to replication stress.

## Introduction

DNA lesions can cause stalling and collapse of the replication fork and lead to chromosome breaks, mutations, genome rearrangements and eventually cancer [1]. To prevent this, a replication checkpoint has evolved as surveillance mechanism to control components of the replisome [2] and to allow coordinating replication with cell cycle progression and DNA repair. Maintenance of stable replication intermediates when DNA synthesis is impeded, requires replisome stability and checkpoint-dependent phosphorylation [3]. Although crucial targets for this checkpoint function await identification, nuclease activities are particularly likely to require fine-tuning, to avoid unscheduled DNA processing under DNA replication stress [4].

Exo1 is a Rad2 family DNA repair nuclease able to remove mononucleotides from the 5′ end of the DNA duplex [5] that was originally identified in the *Schizosaccharomyces pombe* [6] and subsequently in humans [7]. Exo1 is implicated in several DNA repair pathways including mismatch repair, post replication repair, meiotic and mitotic recombination and double strand break repair [8]–[12]. *Saccharomyces cerevisiae* Exo1 acts redundantly with Rad27 in processing Okazaki fragments during DNA replication [13]. More recently, Exo1 was shown to be recruited to stalled replication forks where it counteracts fork reversal [4]. Human EXO1 activity is controlled by post-translational modifications, with ATR-dependent phosphorylation targeting it to ubiquitin-mediated degradation upon replication fork stalling [14], [15], and ATM-dependent phosphorylation apparently restraining its activity during homologous recombination [16]. Analogously, Mec1-dependent phosphorylation inhibits yeast Exo1 activity at uncapped telomeres [17]. Studies in budding yeast showed that *EXO1* deletion suppresses the sensitivity of *rad53*, but not *mec1*, mutant cells to agents causing reversible or irreversible stalling of replication forks [18]. Taken together, this evidence indicates that Exo1 activity is tightly controlled under DNA replication stress and DNA damage.

Eukaryotic 14-3-3 are highly conserved proteins that establish phosphorylation-dependent interactions and modulate the functions of proteins involved in processes such as metabolism, protein trafficking, signal transduction, apoptosis and cell-cycle [19]. Seven 14-3-3 isoforms exist in mammalian cells, but only two in yeast. Structural analysis showed that 14-3-3 proteins self-assemble into flexible homo- and hetero-dimers forming a central groove that is able to adapt two extended peptides [20], [21]. This feature confers them the ability to act as adaptors that integrate signals from different pathways [22], [23]. 14-3-3 proteins can also bind cruciform DNA [24] and replication initiation proteins such as Mcm2 and Orc2 [25]. Upon DNA damage and DNA replication stress, 14-3-3 proteins are required for cell cycle restart, suppression of genomic instability and viability [26]. Moreover, 14-3-3 proteins genetically and physically interact with the checkpoint protein Rad53 [27] as well as the acetyltransferases and deacetylases Esa1 and Rpd3 upon replication perturbations [28]. Although these data point to an important role of 14-3-3 during replication stress, the exact mechanism of 14-3-3 action remains unknown.

In this study, we identify 14-3-3 as novel interaction partners of Exo1 and demonstrate that they regulate phosphorylation of the nuclease. We provide evidence for an accumulation of Exo1-dependent ssDNA gaps at stalled forks in yeast 14-3-3 deficient cells and we show that this causes persistent checkpoint activation and recovery defects. We also show that 14-3-3 proteins control progression and stability of replication forks under conditions of limiting nucleotide availability. Taken together, our data demonstrate that 14-3-3 have a crucial role in regulating the function of proteins at stalled forks, among which Exo1 is a key target.

## Results/Discussion

### 14-3-3 proteins interact with EXO1

To identify novel interaction partners for human EXO1 we designed a yeast two-hybrid screen with GAL4-bait fusion proteins that contain either full-length EXO1 or ΔN-EXO1 (EXO1_366–846_), which lacks the entire catalytic domain. Since the former was not expressed (data not shown), we used the latter to screen a blood peripheral cDNA library. Three 14-3-3 proteins were the highest hits (Table S1), with the β- being more represented than the ε- and ζ-isoform. The presence of an established EXO1 binding protein among the hits, MLH1 (Table S1), confirmed the reliability of this screen.

**Figure 1:**
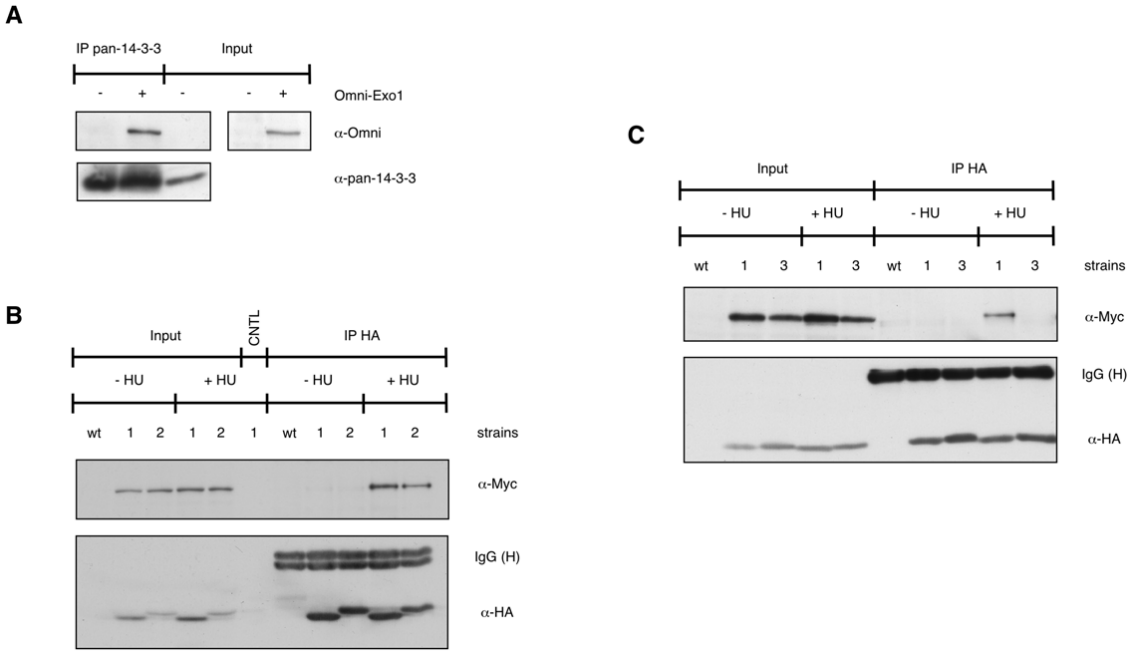
EXO1 interacts with 14-3-3 proteins. (A) HEK-293 cells were transiently transfected with empty vector (−) or pcDNA3.1-Omni-EXO1 (+). Whole cell extracts (WCE, 2.5 mg) were immunoprecipitated with a pan-14-3-3 antibody and proteins were detected as indicated. Input = 50 µg WCE. (B) Control yeast culture (wt) or cultures expressing Bmh1-HA Exo1-Myc (1) or Bmh2-HA Exo1-Myc (2) were treated for 90 min with 150 mM HU. WCE (10 mg) were immunoprecipitated with the monoclonal antibody to HA, proteins were resolved on an 8% SDS-polyacrylamide gel and detected as indicated. CNTL = immunoprecipitation performed in the absence of the antibody. Input = 100 µg WCE. (C) WCE from control yeast culture (wt) or cultures expressing Bmh1-HA Exo1-Myc (1) or Bmh1-280-HA Exo1-Myc (3) were immunoprecipitated as described in (B).

To independently verify these data, we performed co-immunoprecipitation experiments. Given the low abundance of EXO1 in the cell [14], we transiently transfected HEK-293 cells with an Omni-tagged EXO1 construct [14] and immunoprecipitated the expressed protein using a pan-14-3-3 antibody. The data showed that Omni-EXO1 and 14-3-3 proteins could be recovered as a complex (Figure 1A).

To assess the physiological significance of the EXO1/14-3-3 interaction we selected *Sacc. cerevisiae*, a system where only two 14-3-3 proteins are present, namely Bmh1 and Bmh2. In preliminary experiments we examined whether yeast Exo1 and 14-3-3 proteins interact. A C-terminal Myc- or a HA-tag was added to the endogenous *EXO1* or *BMH1/BMH2* genes, respectively. Immunoprecipitation experiments showed that Exo1 formed complexes with Bmh1 or Bmh2 in a HU-dependent manner (Figure 1B). We next explored a possible direct Bmh/Exo1 interaction by Far Western blot analysis. Exo1-Myc immunoprecipitated from control or HU-treated cells was resolved by SDS-PAGE and denatured/renatured on PVDF. Probing the membrane with purified GST-Bmh1 revealed that a direct interaction with Exo1 occurred both in the case of untreated and HU-treated cells (Figure S1). These data possibly indicate that an Exo1 domain normally hidden in non-treated cells may become available for interaction with 14-3-3 proteins upon HU-treatment. Such domain may be artificially exposed during Far Western analysis due to incomplete renaturation of Exo1.

Taken together, these data suggest that the EXO1/14-3-3 interaction is conserved from yeast to mammalian cells. While the interaction is HU-independent in mammalian cells, it requires HU in yeast. This may reflect the different modes of EXO1 regulation in the two systems [15], [17].

### 14-3-3–deficient cells cannot restart stalled replication forks, but their recovery defect is partially suppressed by *EXO1* deletion

**Figure 2:**
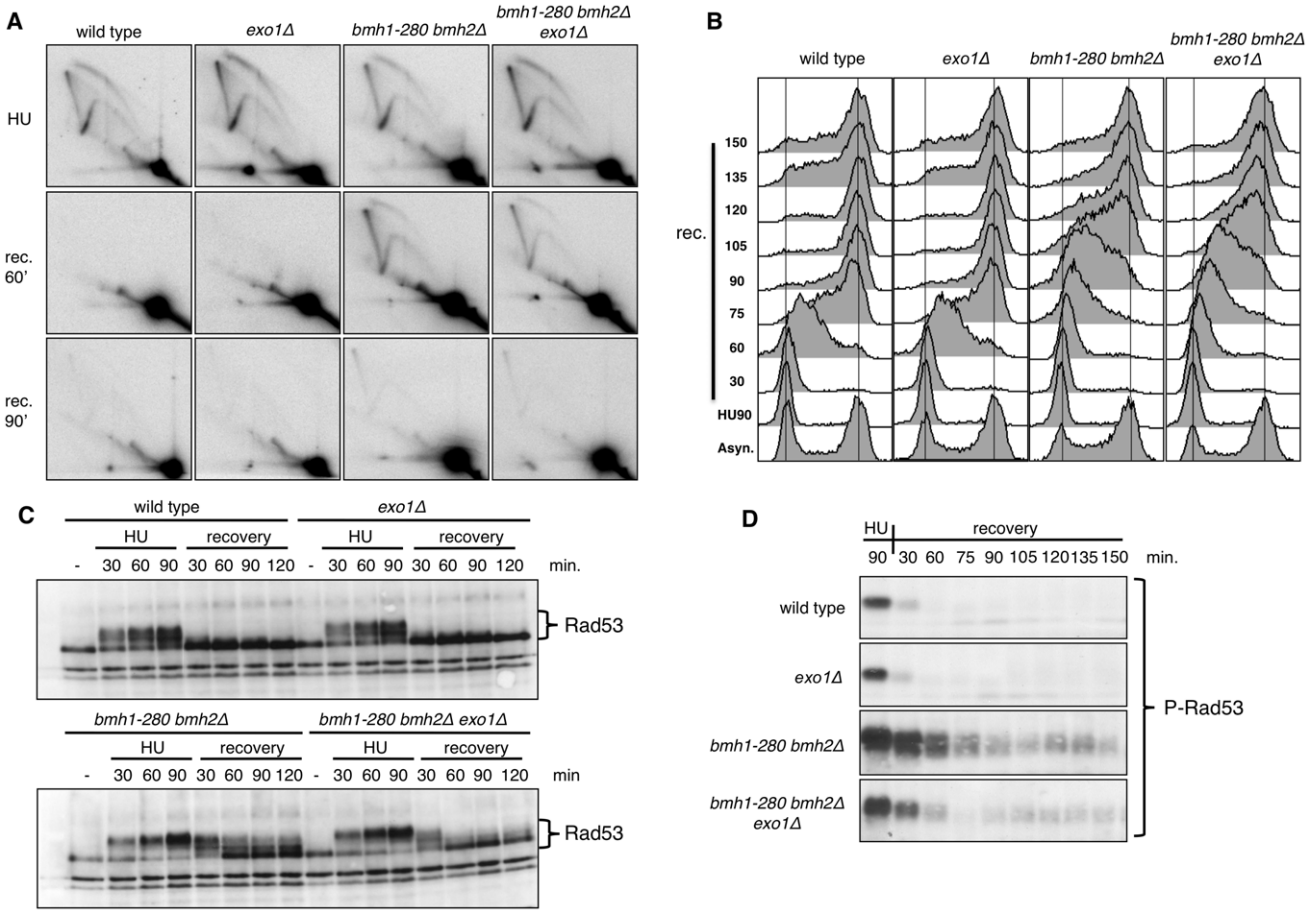
Pattern of HU recovery in wild-type, *exo1Δ*, *bmh1-280 bmh2Δ*, and *bmh1-280 bmh2Δ exo1Δ* strains. (A) Representative 2D gels of replication intermediates (RIs) at ARS305 analyzed after 90 min HU-treatment and upon HU removal (recovery, 60 and 90 min). (B) Time-course flow cytometric analysis of the DNA content in the indicated strains upon recovery from a HU-arrest. (C) Western blot analysis showing in the indicated strains the phosphorylation-dependent mobility shift of Rad53 during the HU-arrest and the recovery phase. (D) Western blot analysis performed with monoclonal antibody F9 to phosphorylated Rad53 showing in the indicated strains the status of Rad53 activation in HU-arrested cells and during the recovery phase.

Genetic and flow cytometric analysis evidenced the sensitivity of 14-3-3-deficient cells to DNA replication stress, with distinct *bmh1* (*bmh2Δ*) alleles showing different defects upon nucleotide depletion (HU) or treatment with DNA damaging agents (UV or methylmethansulfonate, MMS) [26]. However, despite the evidence that 14-3-3 proteins bind origins of replication and cruciform DNA [29], suggesting a regulatory role in DNA replication [25], the issue of possible direct involvement of 14-3-3 in fork stability or processing under genotoxic stress conditions remained to be clarified. Given the comprehensive molecular characterization of yeast Exo1 as component of the replisome and of its role, in checkpoint defective cells, in the processing of forks stalled by nucleotide depletion [4], we focused our investigations on the *bmh1-280 bmh2Δ* double mutant (*bmh1bmh2* hereafter). This mutant shows normal cell cycle progression in unperturbed conditions, but selective sensitivity and cell cycle recovery defects in response to HU [26]. The mutant Bmh1-280 protein carries a single point mutation (E_136_>G) in helix αE at a residue neighboring amino acids that form salt bridges and hydrogen bonds with the ligand [21]. Interestingly, immunoprecipitation experiments showed that the mutant Bmh1-280 protein did not interact with Exo1 in untreated nor HU-treated cells (Figure 1C). Thus, we asked whether the cell cycle recovery defects of this mutant reflect a direct role of 14-3-3 proteins at replication forks and whether Exo1 is also implicated in these processes. We performed neutral-neutral bidimensional gel electrophoresis (2D gel) on the early origin of replication ARS305, which is known to be activated in HU-treated cells [3]. Although the 2D gel pattern looked normal in HU-treated *bmh1bmh2* cells, we observed that replication intermediates (RIs) in 14-3-3 defective cells were still present close to the origin 60 min after HU removal and were only restarted at 90–120 min (Figure 2A and data not shown). This suggests that misregulation of the replisome, without dramatic physical processing of the forks, might be sufficient to impair fork restart. This effect was not detectably suppressed by *EXO1* deletion (Figure 2A).

Flow cytometric analysis of HU-released cells confirmed the slow recovery of the *bmh1bmh2* strain and showed that lack of Exo1 *per se* did not alter the pattern of cell cycle progression (Figure 2B). On the other hand, *EXO1* deletion in a *bmh1bmh2* background led to a partial rescue of the recovery defect, particularly evident at late time points (≥120 min) after release from HU (Figure 2B). This evidence prompted us to ask whether *EXO1* deletion in this background may affect Rad53 activity. Western blot analysis with total and phosphospecific Rad53 antibodies [30] showed that, compared to wild type cells, Rad53 was hyperphosphorylated in HU-treated *bmh1bmh2* cells and that its dephosphorylation was retarded during the HU-recovery phase (Figure 2C and 2D), thus correlating with the described replication restart defect. Importantly, deletion of *EXO1* in 14-3-3-deficient cells re-established to a great extent the pattern of rapid Rad53 dephosphorylation in the recovery phase (Figure 2C and 2D), substantiating the flow cytometry data (Figure 2B).

Overall these data suggest that 14-3-3 proteins are directly implicated in the effective restart of stalled DNA replication forks. Alternatively, they may assist rapid Rad53 dephosphorylation, which is in turn required for fork restart upon HU removal [31]. The latter interpretation is however unlikely as *EXO1* deletion, which markedly restores Rad53 dephosphorylation upon HU removal, does not detectably improve the defective fork restart observed in 14-3-3 deficient cells on the ARS305 replicon. Thus, in the 14-3-3 defective background, Exo1 activity does not directly impact the rate of fork restart, but slows down checkpoint inactivation and delays cell cycle resumption.

### Reversible Exo1 phosphorylation in response to HU is dependent on 14-3-3 proteins

**Figure 3:**
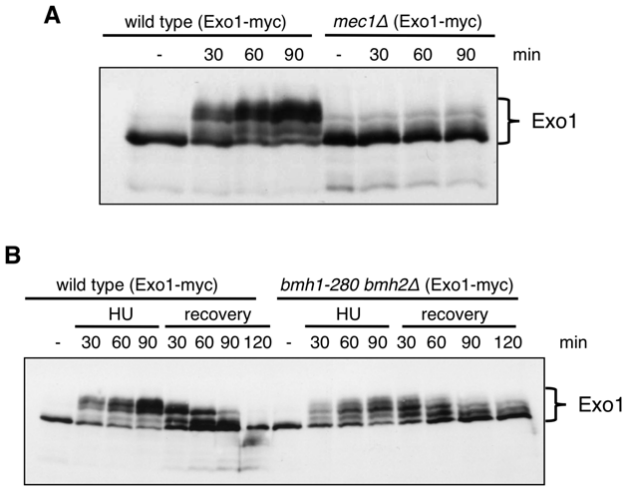
Exo1 phosphorylation pattern in response to HU in wild-type, *mec1Δ*, and *bmh1-280 bmh2Δ* strains. (A) Western blot analysis of Exo1 phosphorylation in HU-arrested cells of wild type and *mec1Δ sml1Δ* strains. (B) Western blot analysis of Exo1 phosphorylation in HU-arrested cells and during the recovery phase of the indicated strains. Both in (A) and (B) proteins were resolved on an 8% Phos-tag SDS-polyacrylamide gel.

Exo1 is controlled in a phosphorylation-dependent manner upon replication fork stalling in mammalian cells [14] and upon a variety of genotoxic insults in yeast [17]. We obtained evidence that yeast Exo1 is phosphorylated in a Mec1-dependent manner also in response to HU (Figure 3A). Notably, the improved resolution of Exo1 phospho-forms by Phos-tag SDS-PAGE [32] allowed us visualizing the complete pattern of Exo1 phosphorylation in response to replicative stress (Figure 3A and 3B).

Next, we asked whether 14-3-3 proteins might be involved in the regulation of Exo1 phosphorylation and stability. Western blot analysis showed that in 14-3-3-deficient cells total Exo1 levels were unchanged (Figure S2), but Exo1 was not phosphorylated to the same stoichiometry observed in wild type cells (Figure 3B, 90 min). Moreover, the rate of Exo1 dephosphorylation upon recovery from HU was considerably reduced in mutant cells, with Exo1 being completely dephosphorylated in wild type but not in 14-3-3-deficient cells (Figure 3B, 120 min). Defective Exo1 phosphorylation in HU-treated 14-3-3-deficient cells is not an indirect consequence of defective checkpoint activation, as under these conditions Rad53, another Mec1-dependent checkpoint target, is promptly phosphorylated (Figure 2C and 2D). Since phosphorylation restrains yeast Exo1 activity [17], we propose that 14-3-3 proteins play an important role in the dynamic control of Exo1 activity upon DNA replication stress and may act as platform for the control of Exo1 phosphorylation. In this respect, attempts to assess the phosphorylation status of Bmh-bound Exo1 were unfortunately inconclusive, as - differently from TCA extracts (Figure 3) - the extracts used for immunoprecipitation fail to be resolved in discrete bands by Phos-tag SDS-PAGE (data not shown). Given that 14-3-3 proteins bind ligands in phospho-dependent and -independent manner [21], it will be important to overcome these technical limitations to address the role of 14-3-3 proteins in controlling the phosphorylation of Exo1 and, possibly, additional targets in the DNA damage response (see below).

### Exo1 is responsible for the accumulation of ssDNA gaps behind the fork in *bmh1bmh2* cells

**Figure 4:**
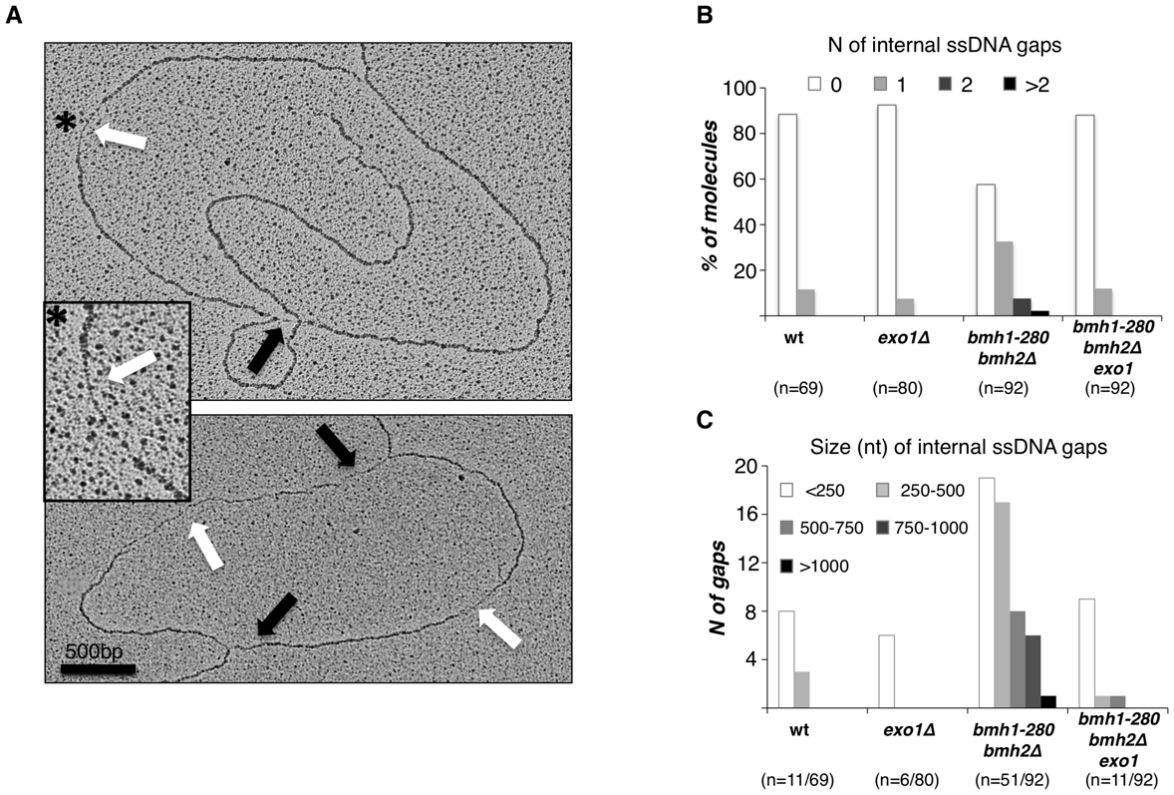
Exo1–dependent generation of ssDNA gaps in *bmh1-280 bmh2Δ* cells. (A) Representative RIs visualized by EM in *bmh1-280 bmh2Δ* cells released synchronously from G1 phase in 0.2 M HU for 1 h: the magnified inset (asterisk) shows a representative ssDNA gap located behind the replication fork. Black arrows: ssDNA gap at the fork; White arrows: internal ssDNA gap located behind the fork. (B) Statistical analysis of ssDNA gap number. The number of analyzed molecules is indicated in brackets. (C) Statistical analysis of ssDNA gap length. The number of analyzed gaps/molecules is indicated in brackets.

As Exo1 activity and Rad53 phosphorylation have been linked to the processing of stalled DNA replication forks, we decided to assess whether defective Rad53 and Exo1 phosphorylation in 14-3-3-deficient cells could reflect changes in the fine architecture of stalled forks. To answer this question, we synchronized the cells in G1, released them for 1 h in YPD medium containing 0.2 M HU and examined RIs by electron microscopy (EM) under non-denaturing conditions [33]. For each strain, about 100 RIs were analyzed in duplicate. 14-3-3-deficient cells showed a dramatic accumulation of ssDNA gaps behind the replication fork (Figure 4A). Statistical analysis indicated that approximately 50% of all RIs analyzed contained one or more ssDNA gaps (Figure 4B). Interestingly, deletion of *EXO1* in the *bmh1bmh2* background completely suppressed this phenotype, leading to a reduction of the ssDNA gaps behind the fork to a level similar to wild type or *exo1Δ* cells (Figure 4B). The comparison of ssDNA gaps length scored by EM evidenced a striking difference: whereas *bmh1bmh2* cells displayed a significant number of large size gaps (>0.5 Kb), the latter were absent in *bmh1bmh2exo1Δ* cells (Figure 4C). The resolution limit of 50–70 nucleotides may have impaired detection of nicks/small gaps in this as well as in previous EM studies with HU [34]. Such structures, however, become visible in 14-3-3-deficient cells, where the unleashed Exo1 activity would enlarge gaps above the detection limit.

These data suggest that 14-3-3 proteins are required to prevent unscheduled Exo1 activity behind stalled replication forks in a checkpoint-proficient background. The implications of these observations are of great significance. A loose control of Exo1 activity may render DNA synthesis more discontinuous in conditions of replicative stress, either promoting additional uncoupling/repriming events or enlarging existing ssDNA gaps via 5′-3′ exonucleolytic processing. Although additional work is required to directly address this point, it is conceivable that continuous polymerase stall due to insufficient deoxynucleotide levels might *per se* lead to increased repriming events, thus raising the number of 5′-ends available for processing by Exo1. In this setting, a strict control of Exo1 activity would be needed to limit damage. We observed no bias for the presence of gaps in leading vs. lagging strands - whenever these could be identified [35] - and we could occasionally detect gaps on opposite strands within the same molecule (Figure S3), suggesting that unscheduled Exo1 activity in 14-3-3 defective cells is not restricted to leading or lagging strand.

### Defective fork progression in 14-3-3–defective cells is independent on *EXO1*

**Figure 5:**
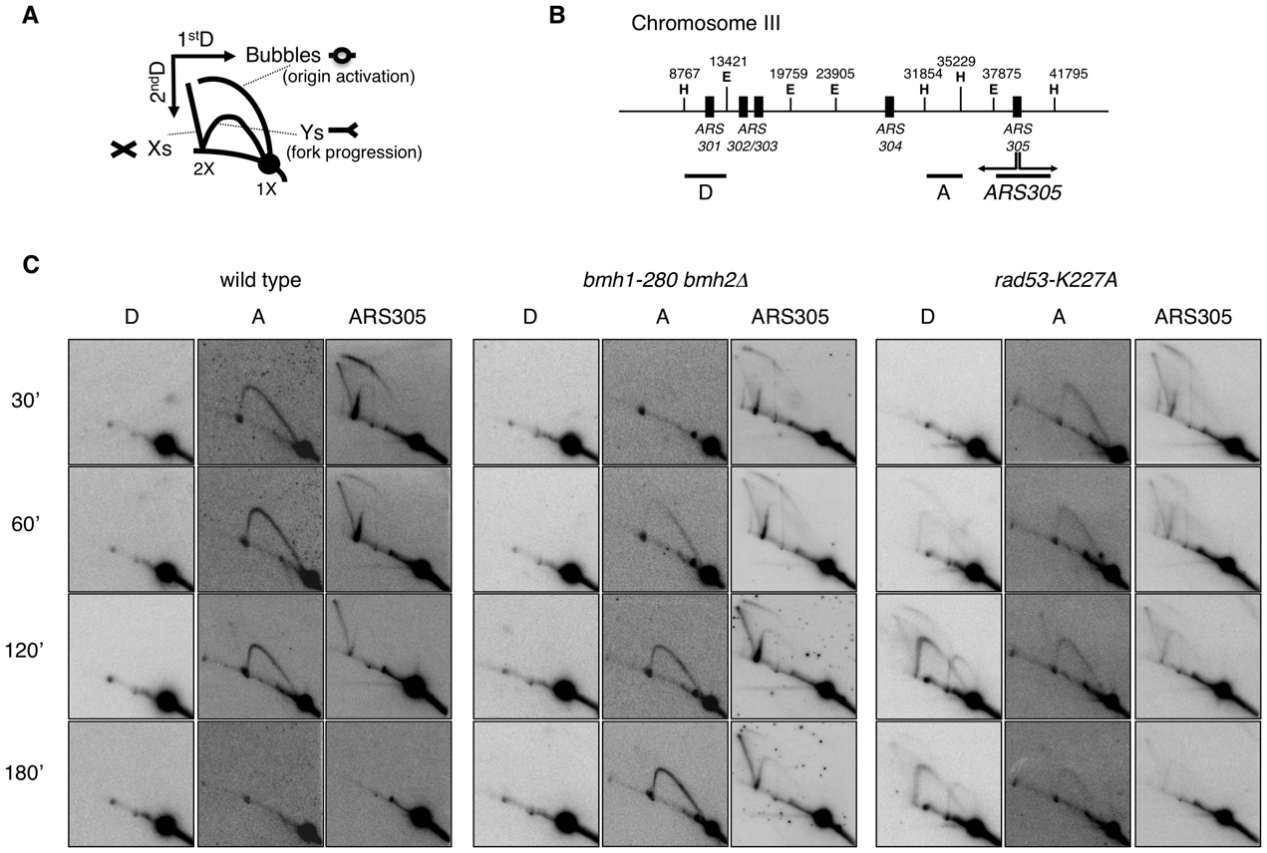
2D gel analysis of RIs from wild-type, *bmh1-280 bmh2Δ*, and *rad53-K227A* strains. (A) Schematic representation of RIs visualized by 2D gel electrophoresis. (B) Chromosome III region adjacent to ARS305 with indication of restriction sites and probes used in 2D gel analysis (E = EcoRV; H = HindIII). (C) Time-course resolution of RIs obtained from the indicated strains arrested in G1 (α-factor) and released in S phase in the presence of 0.2 M HU. Genomic DNA was extracted at the indicated time points and digested with EcoRV and HindIII prior to standard 2D gel analysis.

Replication recovery defects have been previously described and usually reflect replication fork collapse detectable by 2D gel analysis [3]. On the contrary, stalled replication forks in 14-3-3 deficient cells, albeit unable to restart DNA synthesis and abnormally processed by Exo1 activity, upon prolonged HU treatment show a 2D gel pattern indistinguishable from that of wild type cells. We thus decided to investigate in more detail the structure and progression of these forks, performing 2D gel analysis at different time points after HU addition. To this end, cells synchronized in G1 by α-factor were released into medium containing HU and RIs were examined by 2D gels. Figure 5B shows the probes designed to visualize replication fork progression in a region of Chromosome III that contains, besides the early active origin ARS305 [36], a contiguous passively replicated region (Part A) and a region including the dormant origin ARS301 (Part D) [3]. As compared to wild type, *bmh1bmh2* cells showed the same kinetics of origin firing, albeit with slightly lower efficiency as revealed by the intensity of the bubble arc at 30 min (Figure 5C). Progression of the forks in HU from ARS305 across the region of Part A (∼5 Kb to the left of ARS305) was completed after 2–3 h in wild type cells, with the peak of intermediates detectable after ∼1 h. In *bmh1bmh2* cells the first intermediates appeared on this region with 30 min delay, whereas the peak of intermediates was delayed of ∼2 h as compared to wild type cells (Figure 5C), indicative of a significant decrease in the rate of the replication fork progression in HU. Slow RIs disappearance from the origin and delayed invasion of adjacent chromosomal regions may in principle result also from asynchronous firing of ARS305 during the HU arrest. However we consider this alternative interpretation unlikely for the following reasons: a) by budding experiments, 14-3-3 defective cells do not display asynchronous entrance in S-phase (data not shown); b) the comparable intensity of the Y arc on fragment A in the wt (60 min) and in the 14-3-3 mutant (180 min) suggests that forks progress synchronously but slower from the early origin ARS305; c) accordingly, the progressive accumulation of the Y signal on fragment A in 14-3-3 defective cells strictly correlates with the disappearance of the bubble ark on the ARS305 fragment, further suggesting slow but synchronous progression of replication forks on the ARS305 replicon.

It was previously shown that yeast 14-3-3 proteins bind to the checkpoint kinase Rad53 and directly influence its DNA damage-dependent functions [27]. Therefore, we asked whether the slow fork progression in *bmh1bmh2* cells might be solely due to checkpoint defects. To address this issue, we used checkpoint defective Rad53-mutant cells (*rad53-K227A*). The latter displayed striking differences when compared to *bmh1bmh2* cells. Both the destabilization of RIs (ARS305 and Part A) and the uncontrolled firing of dormant origins displayed by *rad53-K227A* cells (Part D) [3], were absent in *bmh1bmh2* cells (Figure 5C). Furthermore, EM did not display any fork reversal or accumulation of ssDNA at replication forks, typical of HU-treated *rad53* cells [34] (data not shown). Finally, 2D gel analysis (Figure S4C, S4F and S4H) and drop assays (Figure S5) revealed synergistic effects of 14-3-3 and Rad53 on both fork stability and survival. Overall, these data indicate that the phenotype observed in 14-3-3 deficient cells reflects a genuine role of 14-3-3 proteins at replication forks and that 14-3-3 and Rad53 have crucial but distinct roles at HU-challenged forks.

Deletion of *EXO1* partially rescued the HU-sensitivity of *rad53-K227A* cells, but not of a *bmh1bmh2* strain (Figure S5). Furthermore, in contrast to checkpoint defective cells, where stability of RIs could be rescued by *EXO1* deletion [4], fork progression defects of *bmh1bmh2* cells were not rescued by loss of *EXO1* (Figure S4G). Thus, while the processing defect that leads to accumulation of ssDNA gaps in 14-3-3-deficient cells was completely suppressed by EXO1 deletion, this did not reflect in suppression of HU sensitivity nor of defective fork progression in HU-treated 14-3-3 deficient cells. Altogether this evidence suggests that 14-3-3 proteins might regulate additional targets during replication stress, possibly through modulation of their phosphorylation. This is not unexpected, given the role of 14-3-3 as integrators of signaling pathways [19] and considering the multiplicity of 14-3-3 targets [37], [38]. Our data implicate 14-3-3 proteins as possible central regulator of the checkpoint response. In analogy with previously reported cases [22] and according to structural data on the dynamic nature of 14-3-3 dimers [39], one may envisage a role for 14-3-3 proteins as docking clamp tethering Exo1 - and other unknown targets - with the kinase controlling its/their activity. Notably, 14-3-3 proteins were reported to bind Rad53 [27], one of the candidate checkpoint kinases required for Exo1 phosphorylation [17].

In conclusion, this work sheds further light on processes occurring at stalled replication forks, proposing 14-3-3 proteins as central integrators of signals that regulate fork stability and processing. Challenges lying ahead consist in the identification of components of the replisome, or proteins controlling them, that may be 14-3-3 targets, as well as in the elucidation of the exact mechanism by which 14-3-3 modulate Exo1 phosphorylation and activity.

## Materials and Methods

### Materials

The antibodies used in this study were: goat polyclonal anti-Omni-probe (M21, sc-499, Santa Cruz Biotechnology); rabbit polyclonal anti-pan 14-3-3 (SA-483, Biomol); mouse monoclonal anti-HA (12CA5, Sigma) and anti-Myc (9E10, Santa Cruz Biotechnology); rabbit polyclonal anti-Rad53 (a kind gift from C. Santocanale, Galway, Ireland); mouse monoclonal F9 to phosphorylated Rad53 [30] (a kind gift of M. Foiani, Milano, Italy).

The chemicals and peptides used in this study were: Hydroxyurea (Sigma); α1-Mating Factor (Sigma).

### *Saccharomyces cerevisiae* strains

The yeast strains used in this study are isogenic to W303-1A (wild type) [40] and are listed in Table 1. All strains have been obtained by one step replacement using the indicated markers and tags that have been generated by PCR. The isolated clones have been verified by colony PCR and Southern Blot or Western Blot.

**Table 1:** List of *Saccharomyces cerevisiae* strains used in this study.

Strain Name	Genotype	Origin	Marker	Tags
W303-1A	*MATa leu2-3,112 trp1-1 can1-100 ura3-1 ade2-1 his3-11,15 [phi+]*	[40]		
CY2034	*MATa rad53-K227A::KANMX4*	[4]	KanMX	
CY5145	*MATa exo1Δ::KANMX6*	[4]	KanMX	
CY5469	*MATa rad53-K227A::KANMX4 exo1Δ::HIS3*	[4]	KanMX/HIS3	
KE2	*MATa bmh2Δ::NAT1 bmh1Δ::HIS3::bmh1-280::LEU2*	This study	NatMX/HIS3/LEU2	
KE4	*MATa bmh2Δ::NAT1 bmh1Δ::HIS3::bmh1-280::LEU2 exo1::URA3*	This study	NatMX/HIS3/LEU2/URA3	
KE7	*MATa bmh2Δ::NAT1 bmh1Δ::HIS3::bmh1-280::LEU2 rad53-K227A::KANMX4*	This study	KanMX/NatMX/HIS3/LEU2	
KE8	*MATa bmh2Δ::NAT1 bmh1Δ::HIS3::bmh1-280::LEU2 exo1::URA3 rad53-K227A::KANMX4*	This study	KanMX/NatMX/HIS3/LEU2/URA3	
KE15	*MATa BMH1-HA::URA3::bmh1 EXO1-Myc::KANMX4::exo1*	This study	KanMX/URA3	HA/Myc
KE16	*MATa BMH2-HA::URA3::bmh2 EXO1-Myc::KANMX4::exo1*	This study	KanMX/URA3	HA/Myc
KE17	*MATa bmh2Δ::KANMX4 bmh1Δ::HIS3::bmh1-280-HA::URA3::LEU2 EXO1-Myc::NAT1::exo1*	This study	KanMX/NatMX/HIS3/LEU2/URA3	HA/Myc
THY AP4	*MATa ura3*, *leu2*, *lexA::lacZ::trp1*, *lexA::HIS3*, *lexA::ADE2*	[46]		Myc
YMG1009	*MATa EXO1-Myc::KANMX4::exo1*	This study	KanMX	Myc
YMG 1197	*MATa bmh2Δ::NAT1 bmh1Δ::HIS3::bmh1-280::LEU2 EXO1-Myc::KANMX4::exo1*	This study	KanMX/NatMX/His3/Leu2	Myc
YLL909	*MATa BMH1-HA::URA3::bmh1*	[28]	URA3	HA
YLL910	*MATa BMH2-HA::URA3::bmh2*	[28]	URA3	HA
YMG1201	*MATa EXO1-Myc::HIS3::exo1*	This study	HIS3	Myc
YMG1215	*MATa mec1Δ::TRP1 sml1Δ::HIS3 EXO1-Myc::HIS3::exo1*	This study	HIS3	Myc

All deletion (Δ) strains lack the entire coding sequence. All strains containing the *bmh1-280* mutation have been generated from strain YLL1090 [26]. The strain KE17 has been generated from DMP4644/4A (M.P. Longhese, unpublished) and is a derivative of YLL1090 [26].

### Yeast two-hybrid screen

The yeast two-hybrid screening was performed with ΔN-EXO1 (EXO1_366–846_) as bait on a cDNA library generated from human peripheral blood mRNA (a kind gift of I. Stagljar, Toronto, Canada) as described previously [41] and using THY AP4 as reporter strain.

### Protein extraction, Western and Far Western blotting, immunoprecipitation

Western blot analysis of yeast proteins was carried out upon TCA extraction [42]. To visualize Exo1, an optimized Phos-tag system (50 µM Phos-tag reagent) was employed according to [32]. Proteins were transferred to nitrocellulose (porablot NCP, 0.45 µm pore size, Machery-Nagel) overnight at room temperature applying constant amperage (200 mA). Far Western blotting [43] was performed using purified recombinant GST-Bmh1 [44] to probe Exo1 that was immunoprecipitated from control or HU-treated yeast cells.

HEK293T cells were grown and lysed as described [14] and protein concentration was determined using the Bio-Rad Protein Assay Reagent (Bio-Rad). Immunoprecipitation of Omni-EXO1 or 14-3-3 proteins from 2.5 mg total cell extracts with specific antibodies was performed as previously described [14].

For immunoprecipitation of yeast proteins, cells were lysed using ice-cold lysis buffer (25 mM Tris-HCl pH 7.4, 15 mM NaCl, 15 mM EGTA, 1 mM NaF, 1 mM Na orthovanadate, 4 mM p-Nitro-Phenyl-Phosphate (pNPP), 0.1% Triton X-100, 1 mM PMSF, complete protease inhibitors cocktail (Roche)). 14-3-3-HA was immunoprecipitated from 10 mg total yeast cell extracts using the monoclonal HA antibody.

### 2D gel electrophoresis and electron microscopy

DNA extraction with the CTAB method and neutral-neutral two-dimensional gel electrophoresis were performed as described [45]. EM analysis was performed as described [33].

## Supporting Information

Figure S1 Far Western blot analysis. Exo1-Myc immunoprecipitated from untreated or HU-treated cells was resolved by SDS-PAGE, proteins were transferred to PVDF and denatured/renatured as described in Materials and Methods. The membrane was probed with purified, recombinant GST-Bmh1 (2 µg) (middle), stripped and reprobed with monoclonal antibody 9E10 to the Myc-tag (top). Wt = control; 1 = Bmh1-HA Exo1-Myc; 2 = Bmh2-HA Exo1-Myc. Ponceau Red (PR) is shown in the lower panel as loading control.
(0.28 MB TIF)

Figure S2 Analysis of Exo1 stability. Western blot analysis of Exo1 during HU-arrest and release of the indicated strains. The extracts used in Figure 3 were loaded on a standard (no Phos-tag) SDS-polyacrylamide gel, where Exo1 appears as one compact band. This allows visualizing stable and similar levels of total Exo1 protein in wild type and *bmh1-280 bmh2Δ* strains during HU-arrest and release. Ponceau Red is shown in the lower panel as loading control.
(0.59 MB TIF)

Figure S3 ssDNA gaps arise on both leading and lagging strands in HU-treated *bmh1-280 bmh2Δ* cells. Two representative replication bubbles visualized by EM in *bmh1-280 bmh2Δ* cells synchronously released from G1 phase in 0.2 M HU for 1 h. The molecules are shown at the same magnification. A scale bar is included in the lower panel. Black arrows: ssDNA gaps at the fork. White arrows: internal ssDNA gap located behind the forks. In the top panel, length measurements show that two internal gaps on opposite replicated duplexes cover the same distance from the replication forks: by definition, one must have resulted from leading strand and the other from lagging strand DNA synthesis. Similarly, in the bottom panel, the two internal ssDNA gaps lay very close to opposite forks on the same replicated duplex, marking by definition opposite strands (leading and lagging) of DNA synthesis.
(7.60 MB TIF)

Figure S4 2D gel analysis of RIs from wild-type and mutant strains. Wild-type (B), *rad53-K227A* (C), *exo1Δ* (D), *rad53-K227 exo1Δ* (E), *bmh1-280 bmh2Δ* (F), *bmh1-280 bmh2Δ exo1Δ* (G), *bmh1-280 bmh2Δ rad53-K227A (H)*, *bmh1-280 bmh2Δ rad53-K227A exo1Δ* (I) strains were used for 2D gel analysis as described in Figure 5. Additional genomic fragments (B and C) were visualized by Southern blot on the same filters, as depicted in panel (A).
(4.18 MB PDF)

Figure S5 HU-sensitivity assay of wild-type and mutant strains. Wild-type, *rad53-K227A*, *exo1Δ*, *rad53-K227A exo1Δ*, *bmh1-280 bmh2Δ*, *bmh1-280 bmh2Δ exo1Δ*, *bmh1-280 bmh2Δ rad53-K227A* and *bmh1-280 bmh2Δ rad53-K227A exo1Δ* cultures were grown exponentially. Serial dilutions (1∶10) were spotted on YPD plates containing different HU concentrations and grown for 3 days before scoring.
(3.12 MB TIF)

Table S1 Identification of novel EXO1 interacting partners by two-hybrid-screen in yeast. List of the most prominent proteins found to interact with human EXO1, with indication of the overall hit representation.
(0.14 MB TIF)
