# Supplementary figures and images for: 14-3-3 Proteins Regulate Exonuclease 1–Dependent Processing of Stalled Replication Forks
Source: PLoS Genet. 2011 Apr 14;7(4):e1001367. doi: 10.1371/journal.pgen.1001367 (PMC3077382; doi:10.1371/journal.pgen.1001367)

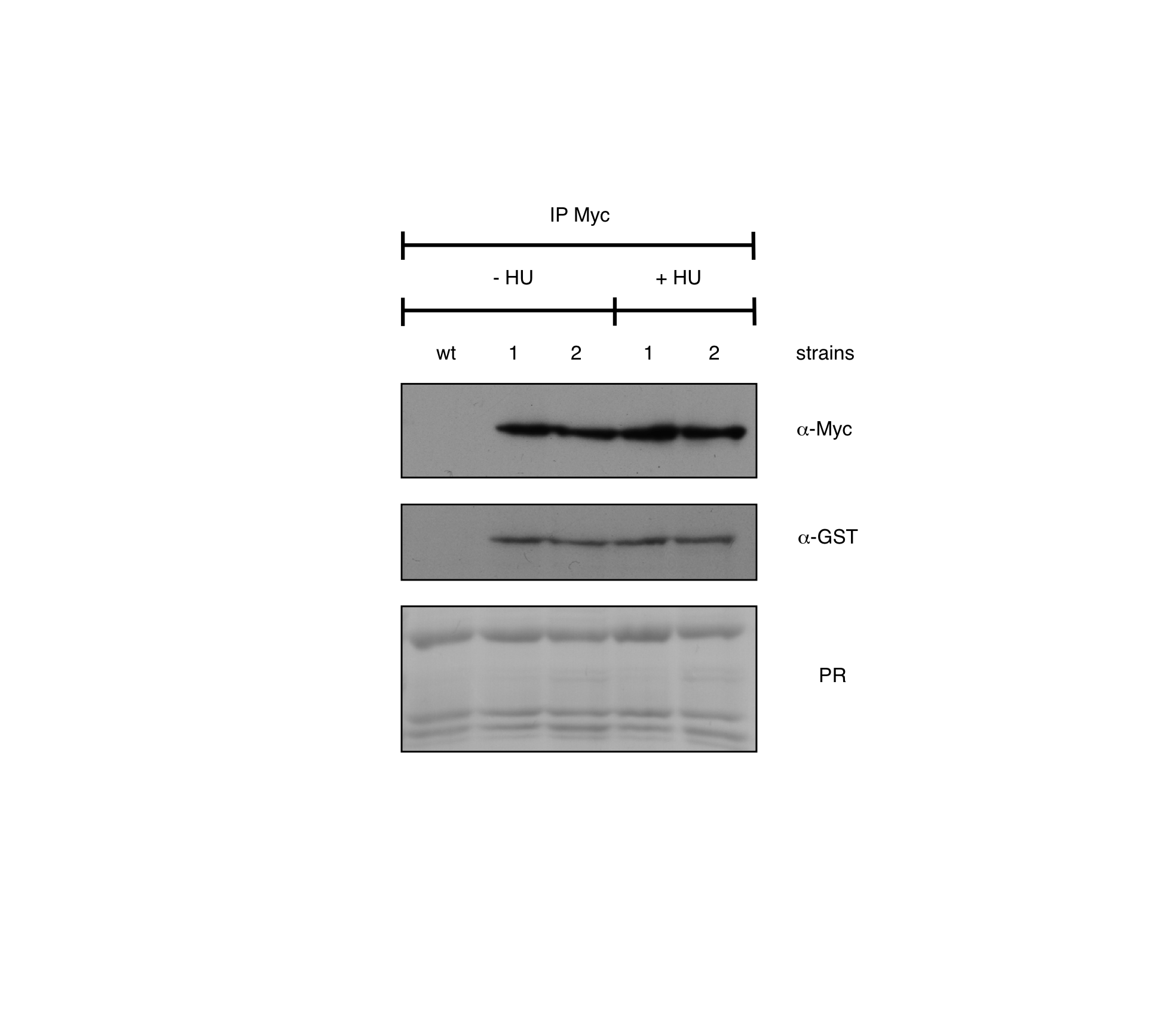

Supplement: Figure S1 — Far Western blot analysis. Exo1-Myc immunoprecipitated from untreated or HU-treated cells was resolved by SDS-PAGE, proteins were transferred to PVDF and denatured/renatured as described in Materials and Methods. The membrane was probed with purified, recombinant GST-Bmh1 (2 µg) (middle), stripped and reprobed with monoclonal antibody 9E10 to the Myc-tag (top). Wt = control; 1 = Bmh1-HA Exo1-Myc; 2 = Bmh2-HA Exo1-Myc. Ponceau Red (PR) is shown in the lower panel as loading control. (0.28 MB TIF) [file pgen.1001367.s001.tif]

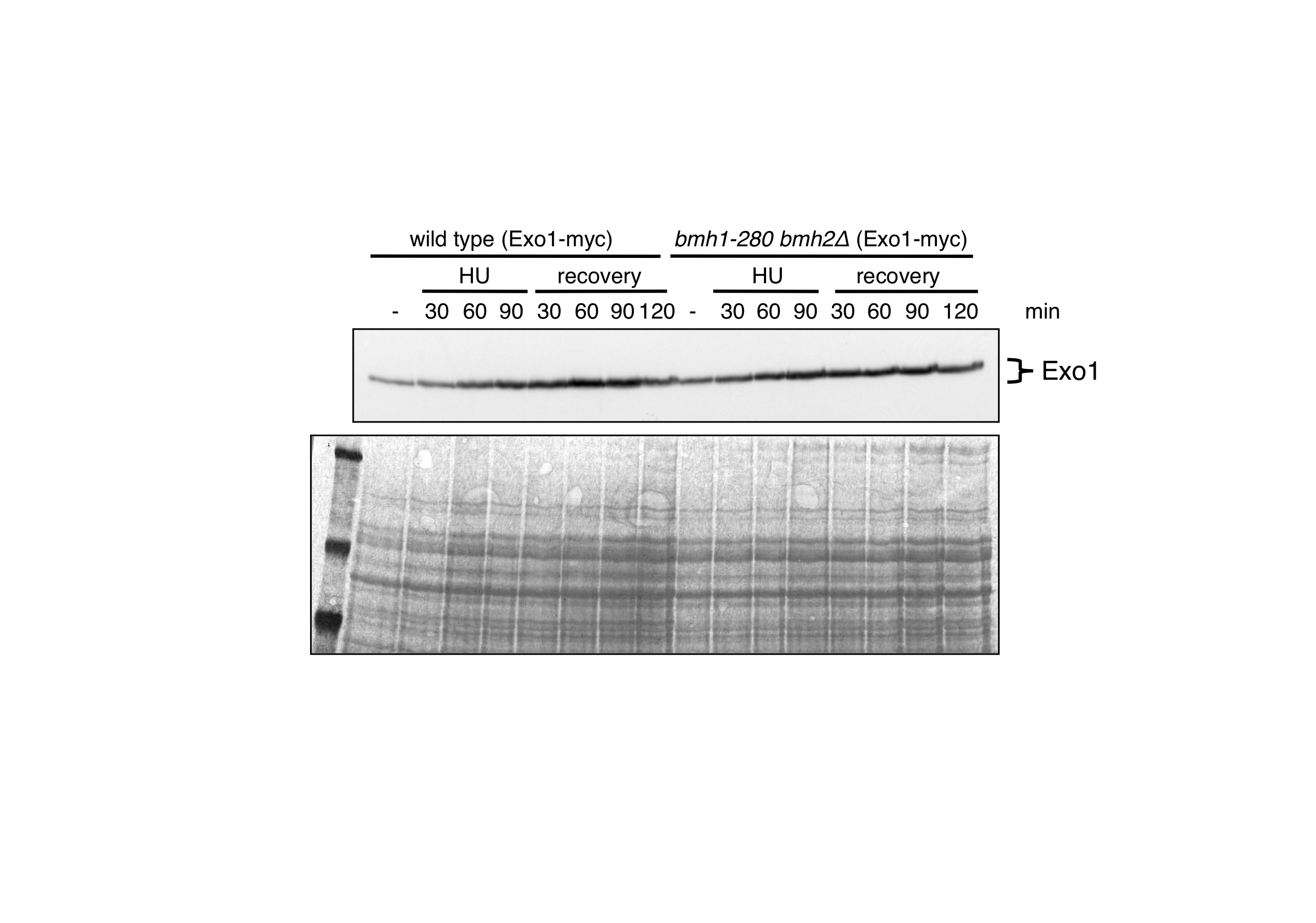

Supplement: Figure S2 — Analysis of Exo1 stability. Western blot analysis of Exo1 during HU-arrest and release of the indicated strains. The extracts used in Figure 3 were loaded on a standard (no Phos-tag) SDS-polyacrylamide gel, where Exo1 appears as one compact band. This allows visualizing stable and similar levels of total Exo1 protein in wild type and bmh1-280 bmh2Δ strains during HU-arrest and release. Ponceau Red is shown in the lower panel as loading control. (0.59 MB TIF) [file pgen.1001367.s002.tif]

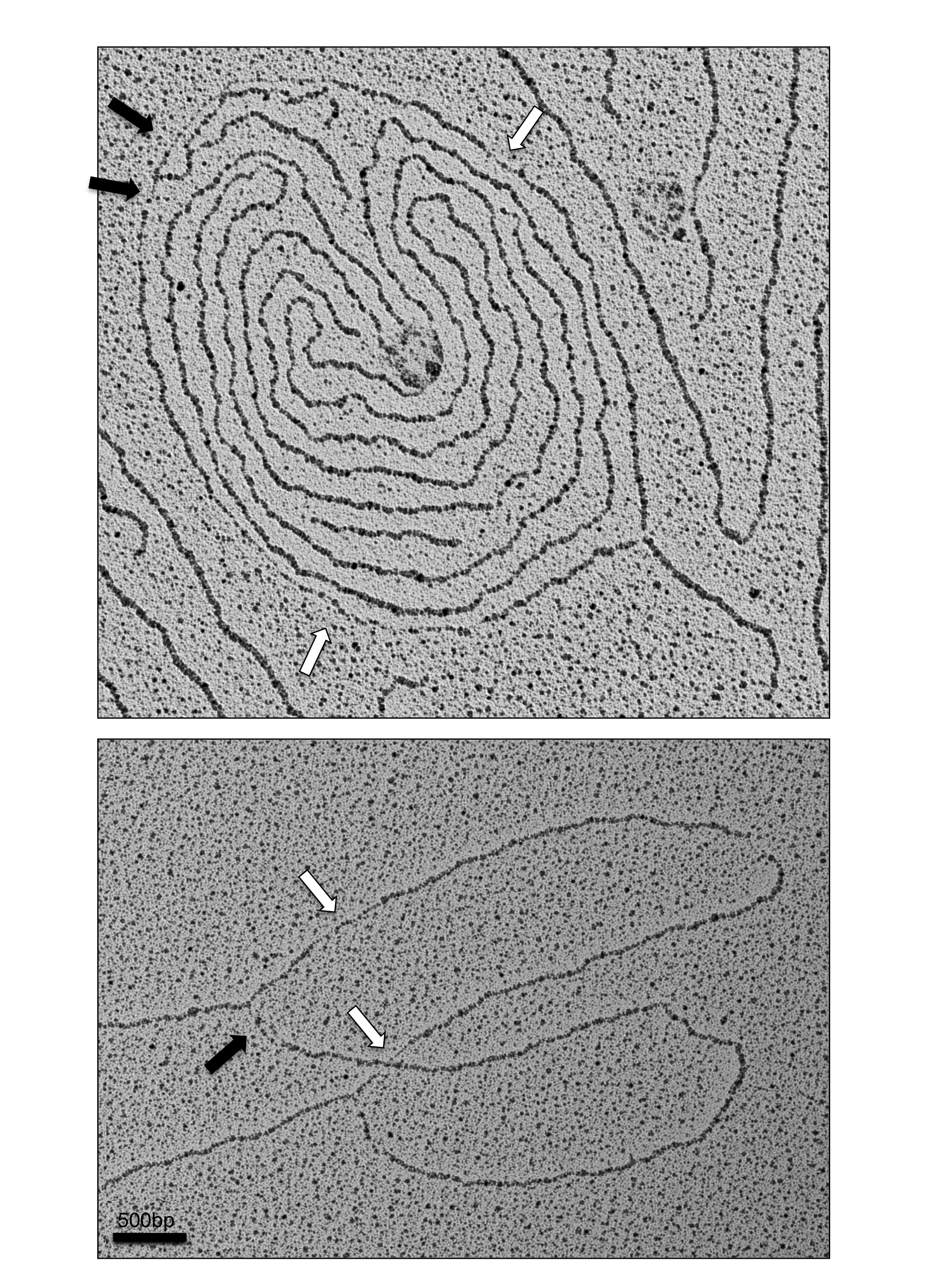

Supplement: Figure S3 — ssDNA gaps arise on both leading and lagging strands in HU-treated bmh1-280 bmh2Δ cells. Two representative replication bubbles visualized by EM in bmh1-280 bmh2Δ cells synchronously released from G1 phase in 0.2 M HU for 1 h. The molecules are shown at the same magnification. A scale bar is included in the lower panel. Black arrows: ssDNA gaps at the fork. White arrows: internal ssDNA gap located behind the forks. In the top panel, length measurements show that two internal gaps on opposite replicated duplexes cover the same distance from the replication forks: by definition, one must have resulted from leading strand and the other from lagging strand DNA synthesis. Similarly, in the bottom panel, the two internal ssDNA gaps lay very close to opposite forks on the same replicated duplex, marking by definition opposite strands (leading and lagging) of DNA synthesis. (7.60 MB TIF) [file pgen.1001367.s003.tif]

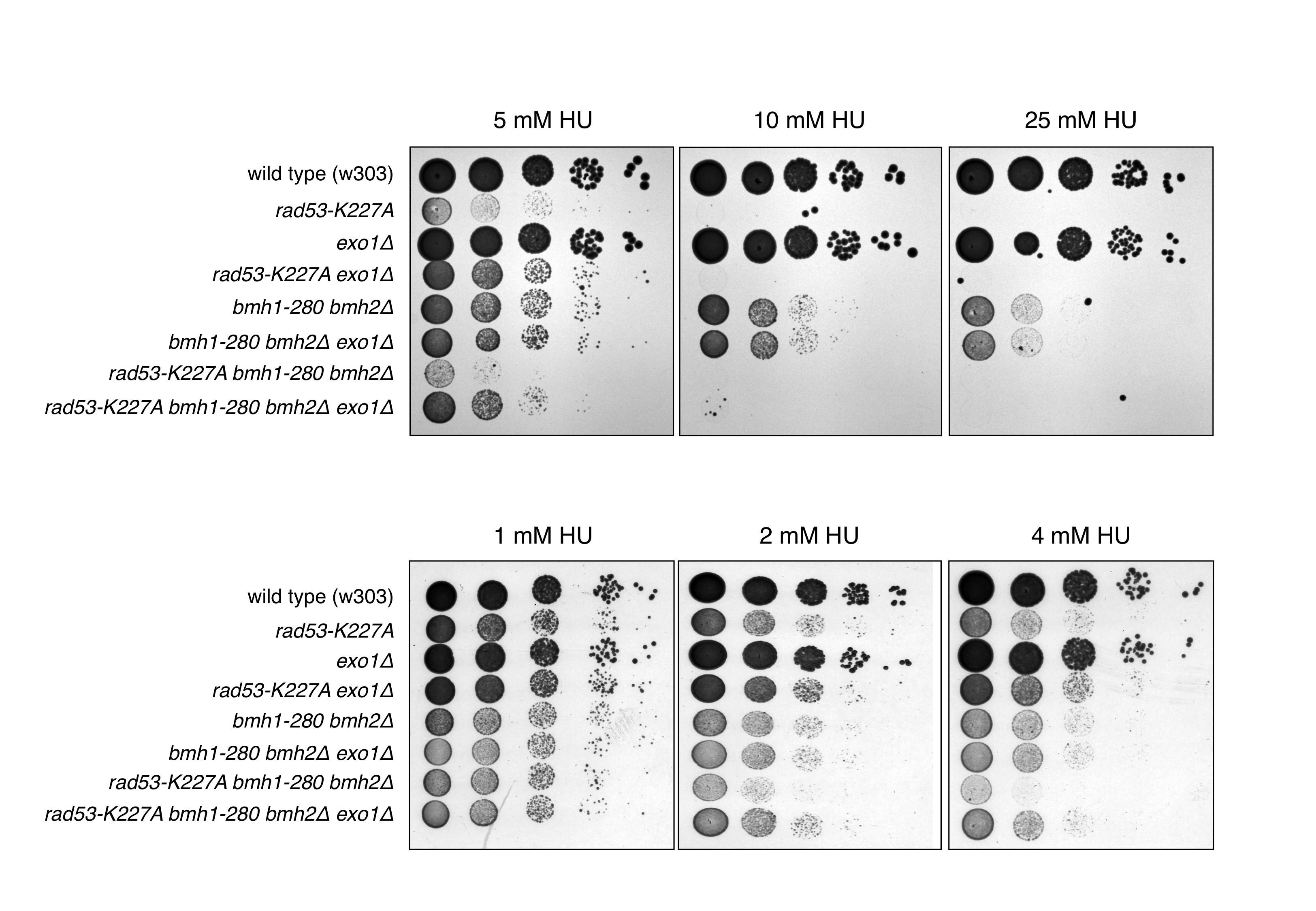

Supplement: Figure S5 — HU-sensitivity assay of wild-type and mutant strains. Wild-type, rad53-K227A, exo1Δ, rad53-K227A exo1Δ, bmh1-280 bmh2Δ, bmh1-280 bmh2Δ exo1Δ, bmh1-280 bmh2Δ rad53-K227A and bmh1-280 bmh2Δ rad53-K227A exo1Δ cultures were grown exponentially. Serial dilutions (1∶10) were spotted on YPD plates containing different HU concentrations and grown for 3 days before scoring. (3.12 MB TIF) [file pgen.1001367.s005.tif]

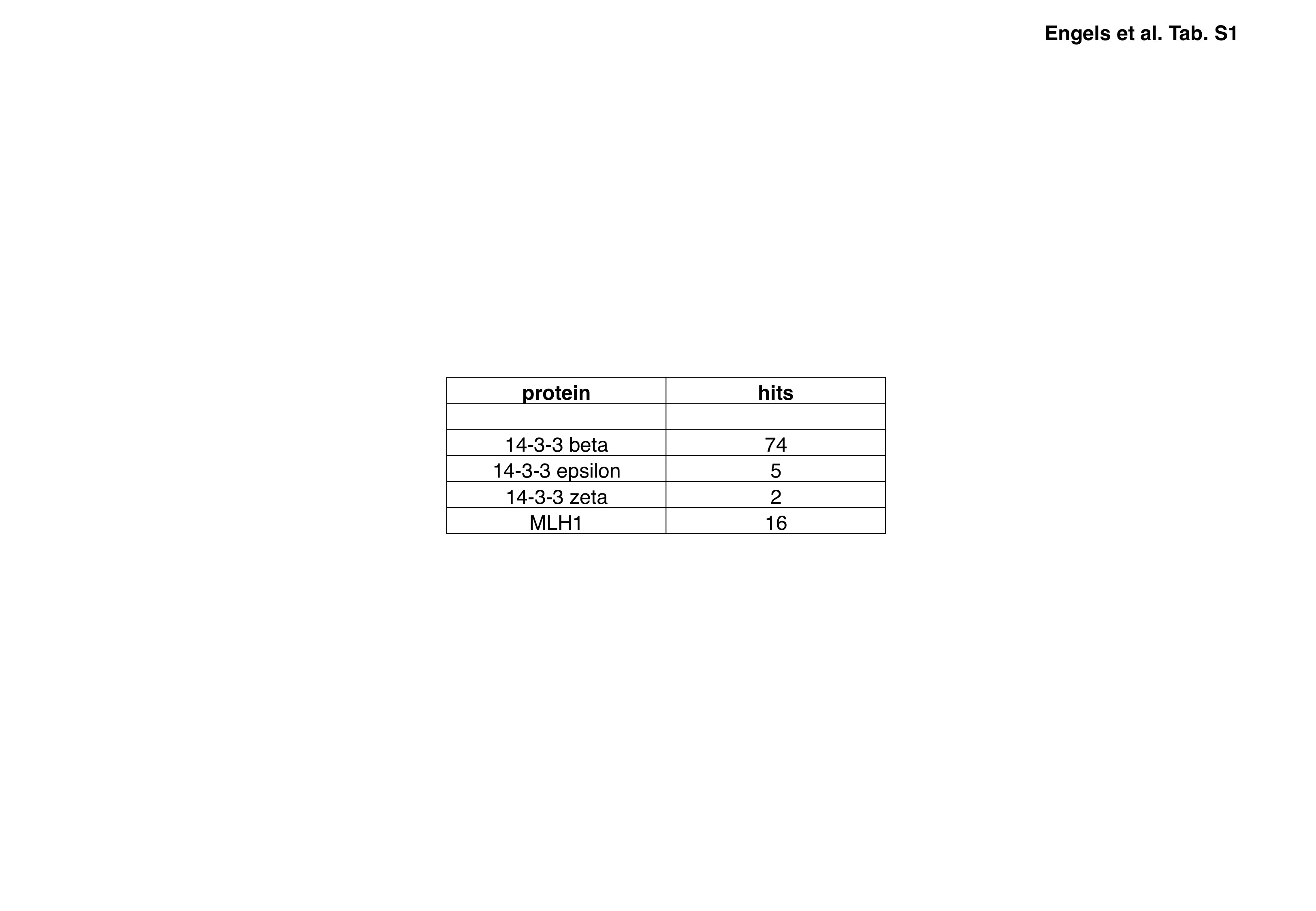

Supplement: Table S1 — Identification of novel EXO1 interacting partners by two-hybrid-screen in yeast. List of the most prominent proteins found to interact with human EXO1, with indication of the overall hit representation. (0.14 MB TIF) [file pgen.1001367.s006.tif]
