# Supplementary material for: 14-3-3 Proteins Regulate Exonuclease 1–Dependent Processing of Stalled Replication Forks
Source: PLoS Genet. 2011 Apr 14;7(4):e1001367. doi: 10.1371/journal.pgen.1001367 (PMC3077382; doi:10.1371/journal.pgen.1001367)

A

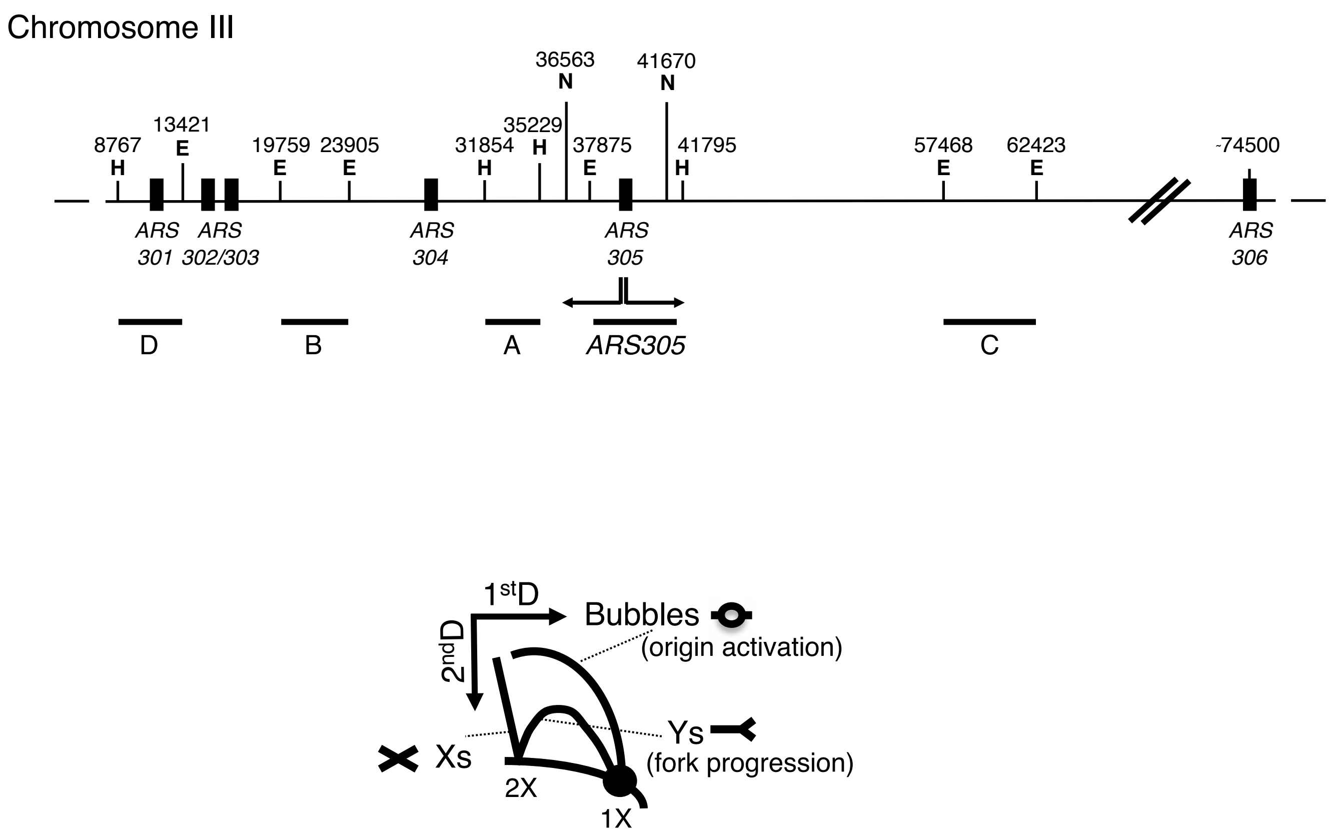
Chromosome III
36563
N
41670
N
13421
E
35229
H
8767
H
19759
E
23905
E
31854
H
37875
E
41795
H
57468
E
62423
E
-74500
ARS 301
ARS 302/303
ARS 304
ARS 305
ARS 306
D
B
A
ARS305
C
1stD
2ndD
Bubbles
(origin activation)
Ys
(fork progression)
Xs
2X
1X

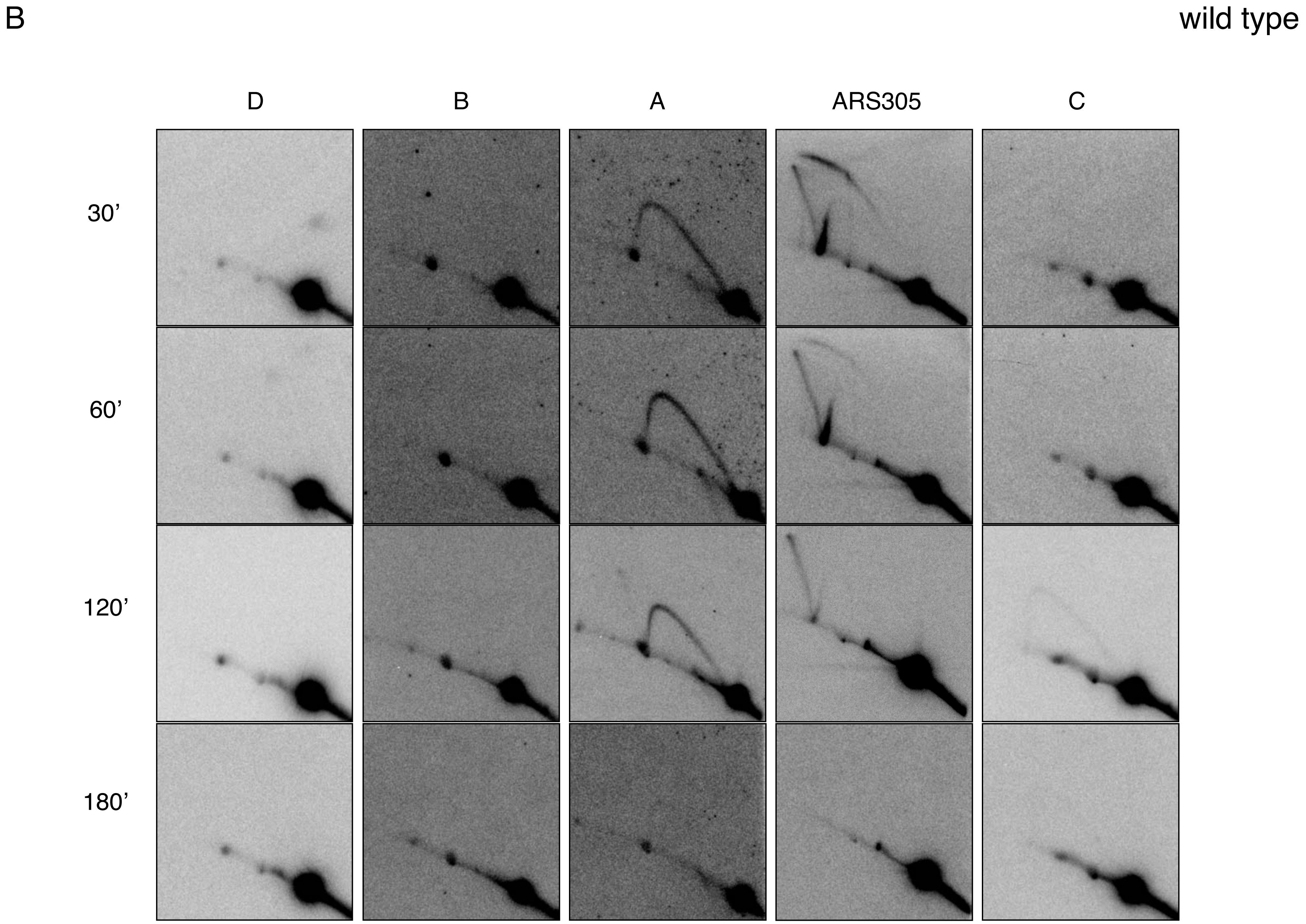

B
wild type
D
B
A
ARS305
C
30’
60’
120’
180’

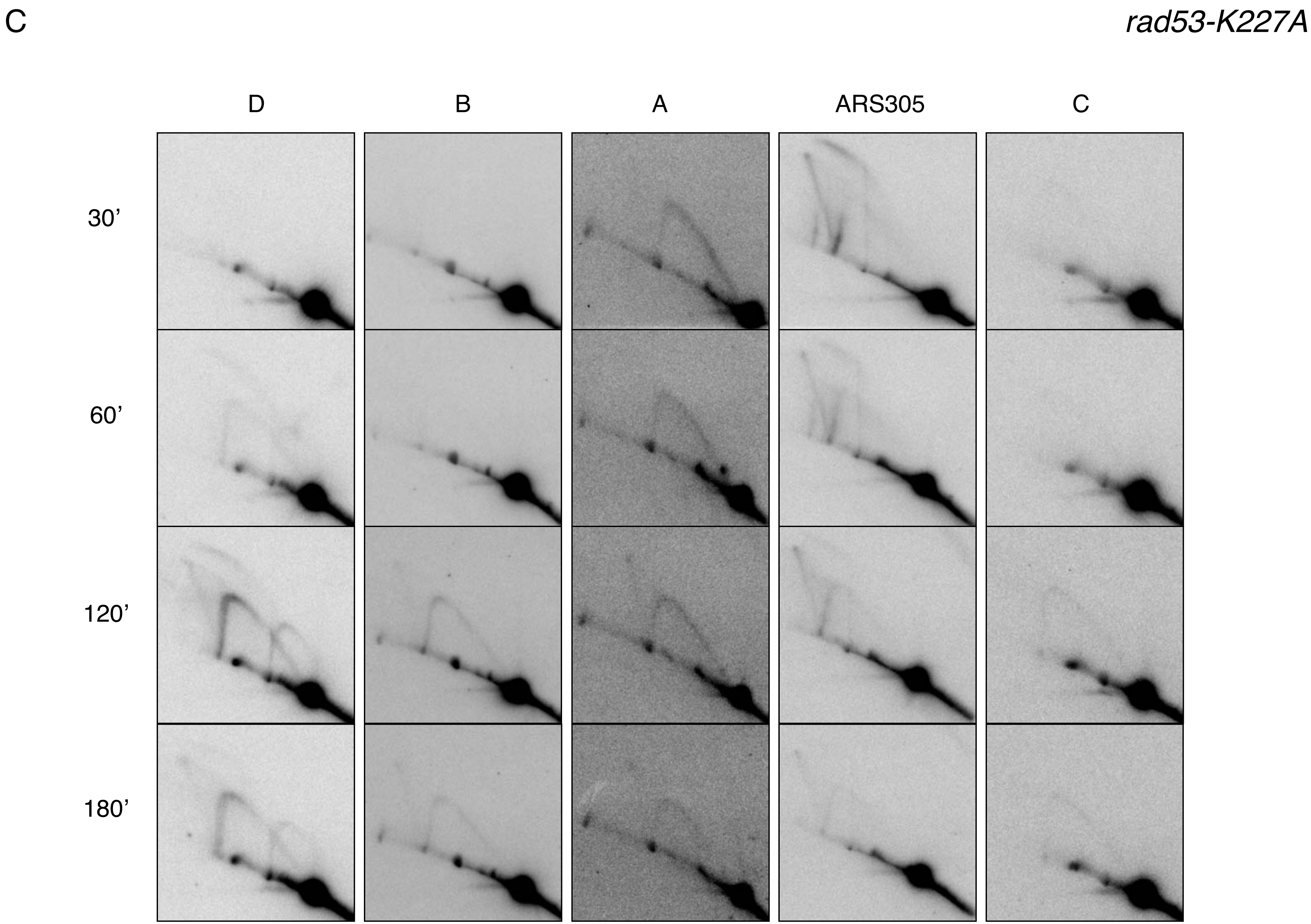
C
rad53-K227A
D
B
A
ARS305
C
30’
60’
120’
180’

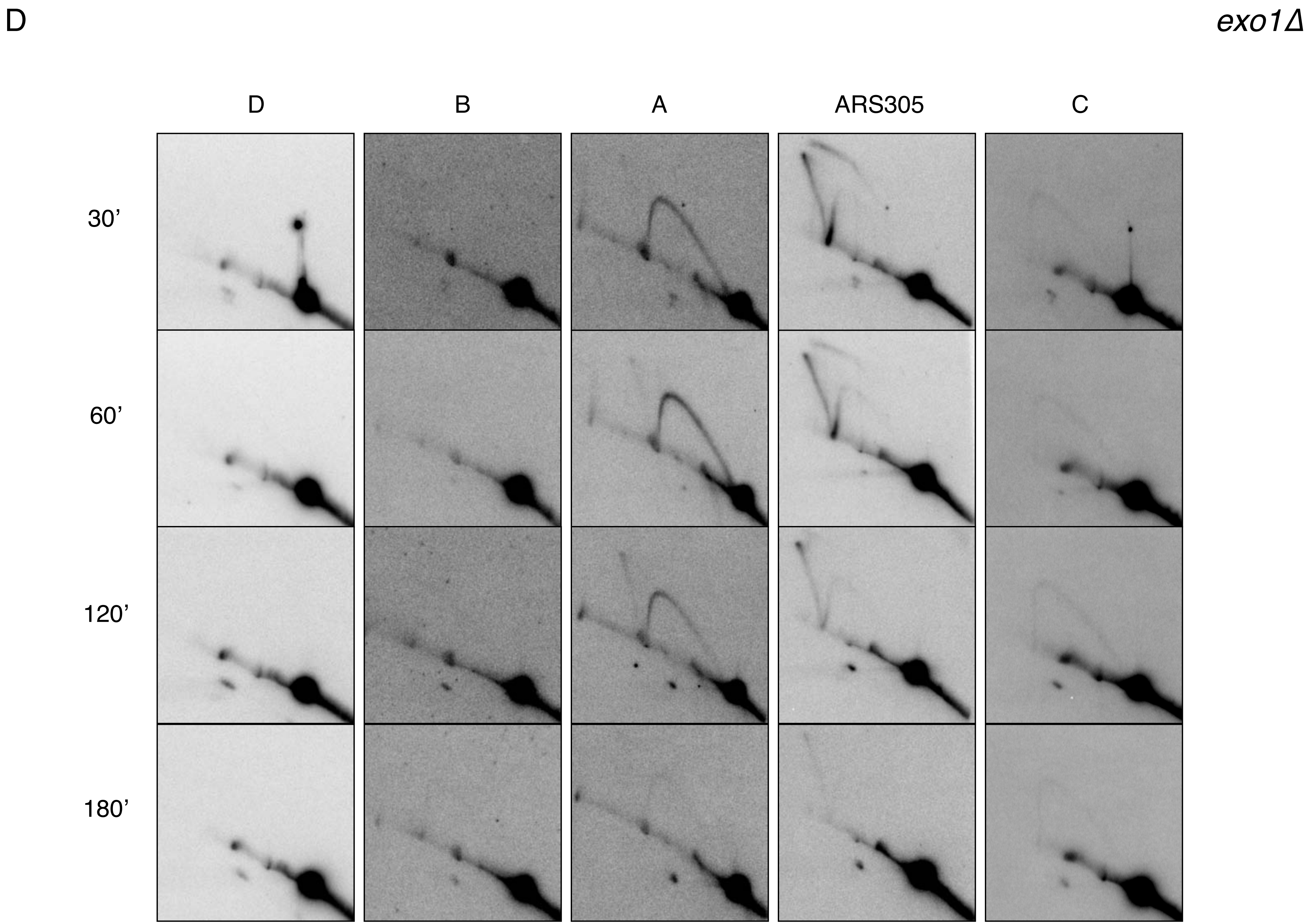
D
exo1Δ
D
B
A
ARS305
C
30’
60’
120’
180’

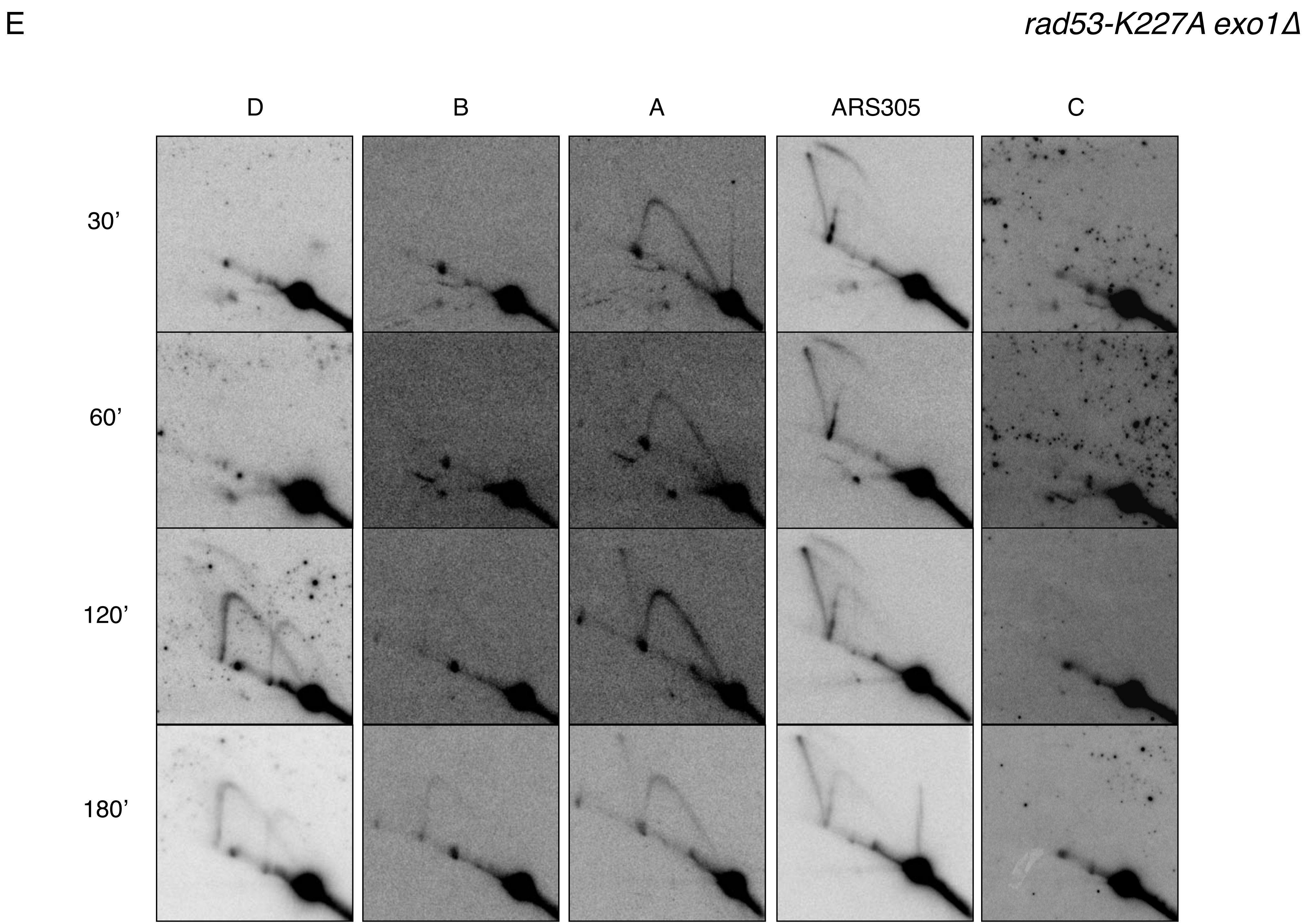
E
rad53-K227A exo1Δ
D
B
A
ARS305
C
30’
60’
120’
180’

F

bmh1-280 bmh2Δ

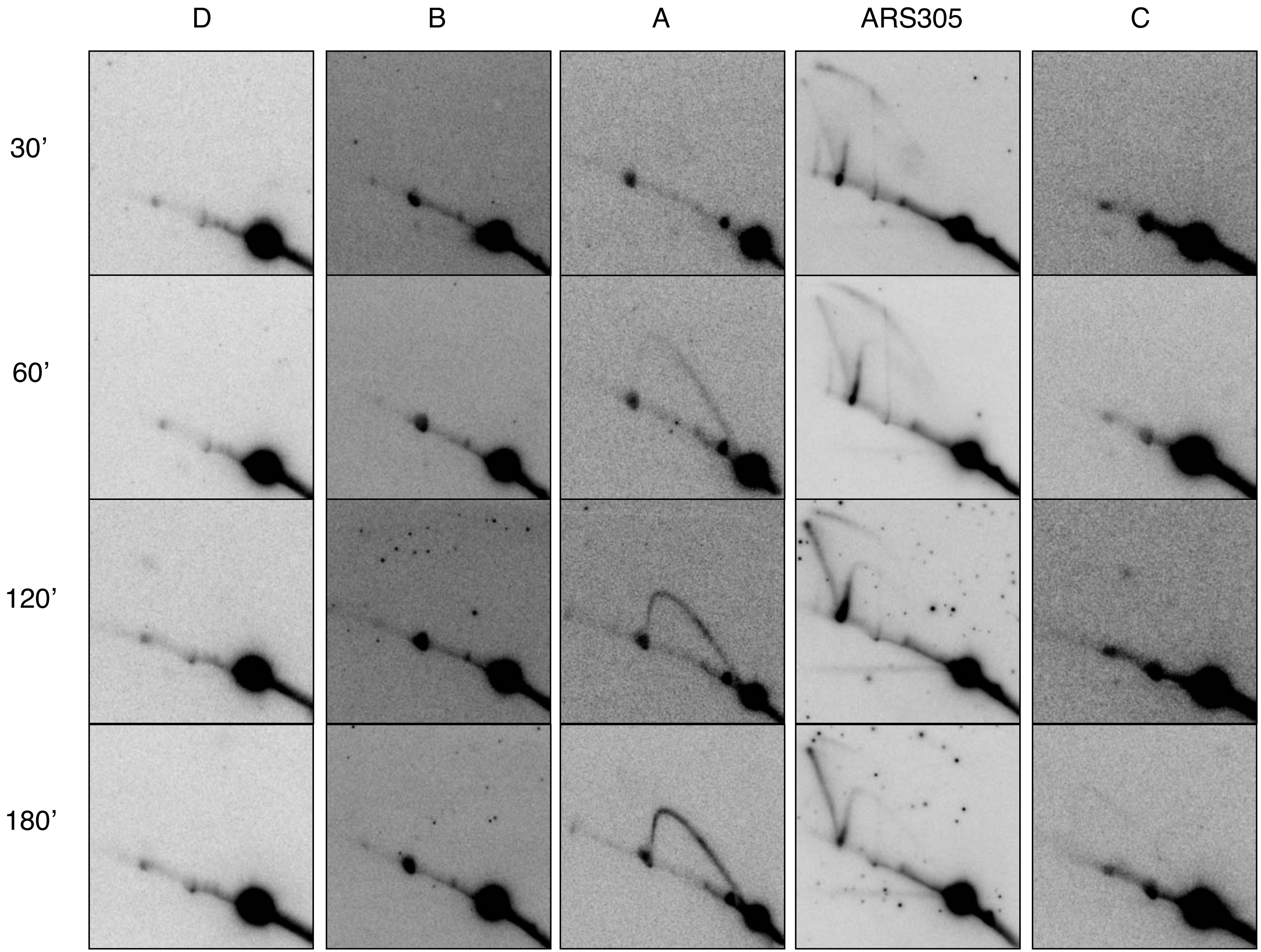

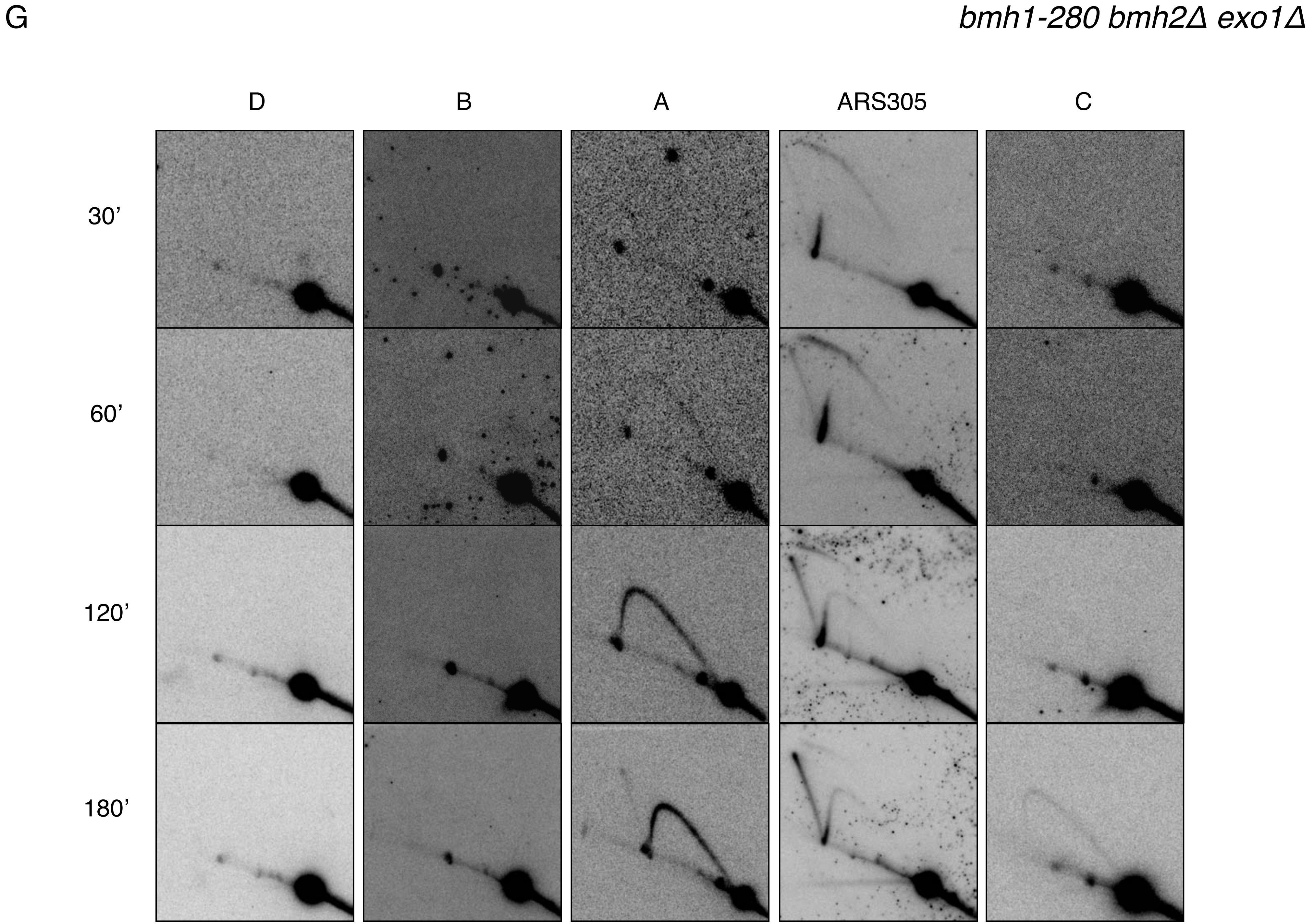
G
bmh1-280 bmh2Δ exo1Δ
D
B
A
ARS305
C
30’
60’
120’
180’

H

bmh1-280 bmh2Δ rad53-K227A

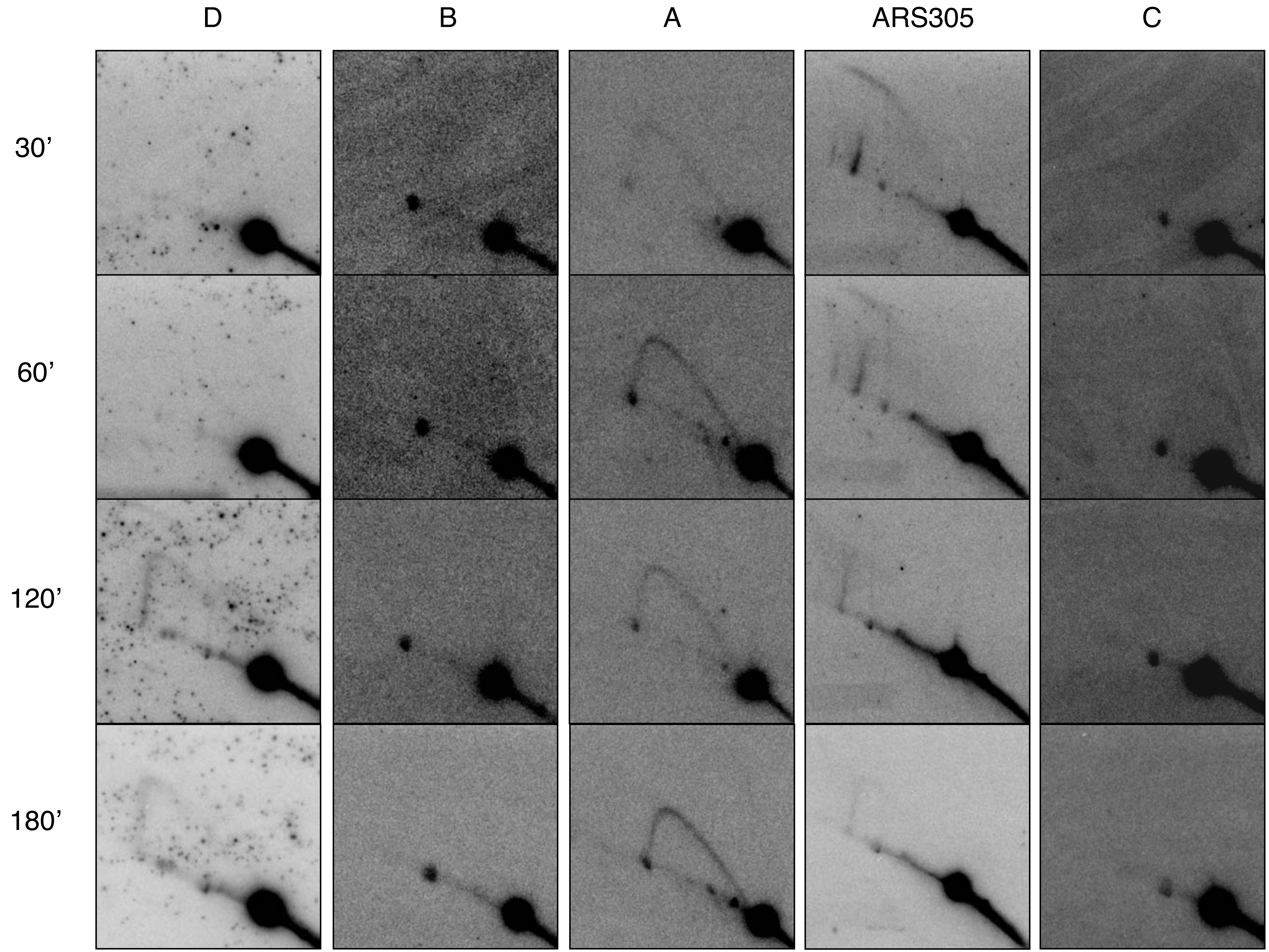

I

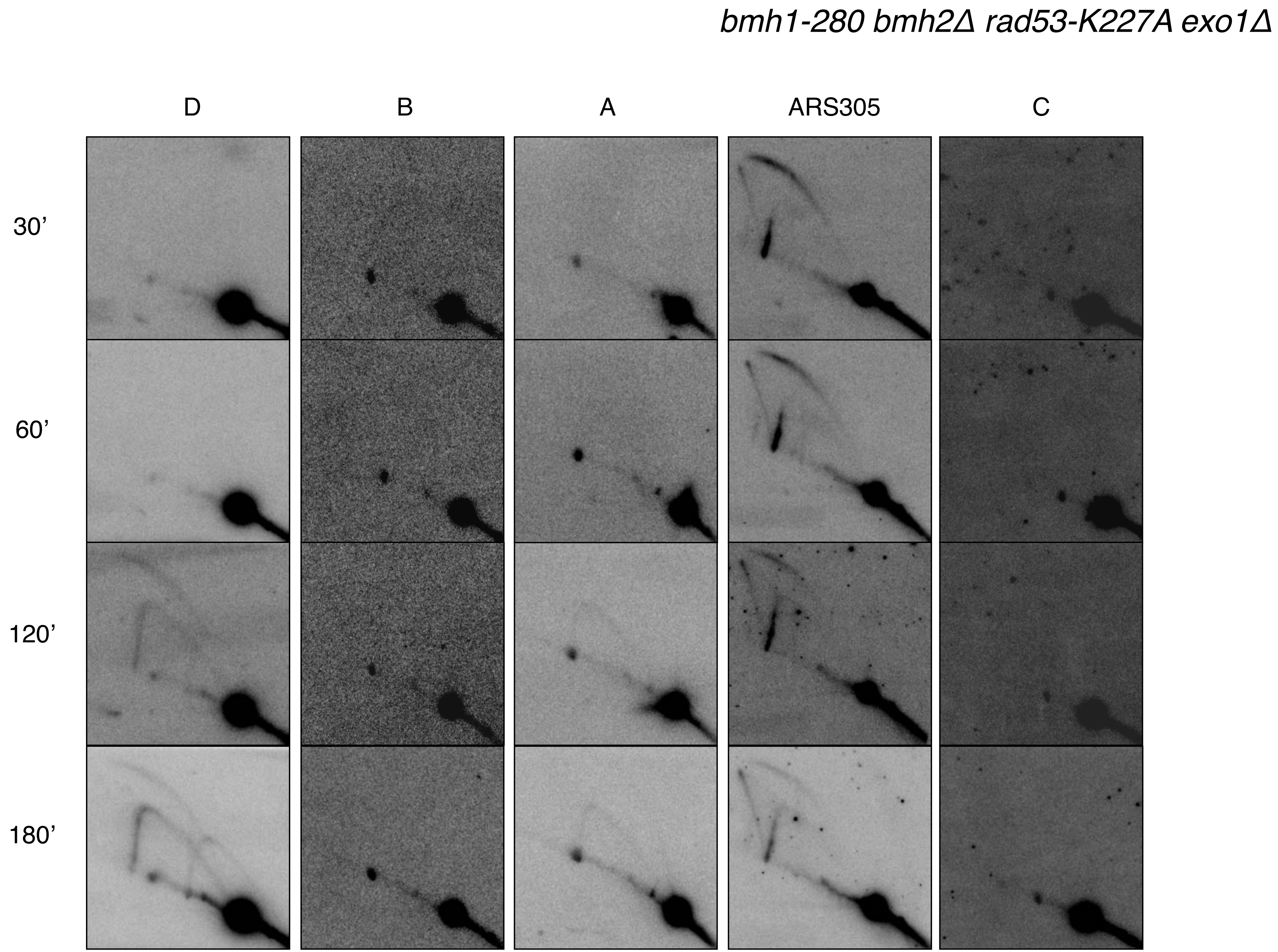

Supplement: Figure S4 — 2D gel analysis of RIs from wild-type and mutant strains. Wild-type (B), rad53-K227A (C), exo1Δ (D), rad53-K227 exo1Δ (E), bmh1-280 bmh2Δ (F), bmh1-280 bmh2Δ exo1Δ (G), bmh1-280 bmh2Δ rad53-K227A (H), bmh1-280 bmh2Δ rad53-K227A exo1Δ (I) strains were used for 2D gel analysis as described in Figure 5. Additional genomic fragments (B and C) were visualized by Southern blot on the same filters, as depicted in panel (A). (4.18 MB PDF) [file pgen.1001367.s004.pdf]
